# Population attributable risk of key modifiable risk factors associated with non-exclusive breastfeeding in Nigeria

**DOI:** 10.1186/s12889-018-5145-y

**Published:** 2018-02-13

**Authors:** Felix Akpojene Ogbo, Andrew Page, John Idoko, Kingsley E. Agho

**Affiliations:** 1 Translational Health Research Institute, School of Medicine, Western Sydney University, Campbelltown Campus, Locked Bag 1797, Penrith, NSW 2571 Australia; 2 Department of Medicine, Faculty of Medical Sciences, University of Jos, Jos, Plateau State P.M.B 2084 Nigeria; 3 Prescot Specialist Medical Centre, Makurdi, Benue State Nigeria; 4 School of Science and Health, Western Sydney University, Campbelltown Campus, Locked Bag 1797, Penrith, NSW 2571 Australia

**Keywords:** Infant, Nigeria, Non-exclusive breastfeeding, Population attributable risk

## Abstract

**Background:**

Non-exclusive breastfeeding (non-EBF) is a risk factor for many of the 2300 under-five deaths occurring daily in Nigeria – a developing country with approximately 40 million children. This study aimed to quantify and compare the attributable burden of key modifiable risk factors associated with non-EBF in Nigeria to inform strategic policy responses and initiatives.

**Methods:**

Relative risk and exposure prevalence for selected modifiable risk factors were used to calculate population attributable fractions based on Nigeria Demographic and Health Surveys data for the period (1999–2013). Scenarios based on feasible impact of community-based interventions in reducing exposure prevalence were also considered to calculate comparative potential impact fractions.

**Results:**

In Nigeria, an estimated 22.8% (95% Confidence Interval, CI: 9.2–37.0%) of non-EBF was attributable to primary and no maternal education; 24.7% (95% CI: 9.5–39.5%) to middle and poor household wealth, 9.7% (1.7–18.1%) to lower number (1–3) and no antenatal care visits; 18.8% (95% CI: 6.9–30.8%) to home delivery and 16.6% (95% CI: 3.0–31.3%) to delivery assisted by a non-health professional. In combination, more than half of all cases of non-EBF (64.5%; 95% CI: 50.0–76.4%) could be attributed to those modifiable risk factors. Scenarios based on feasible impacts of community-based approaches to improve health service access and human capacity suggest that an avoidable burden of non-EBF practice of approximately 11% (95% CI: -5.4; 24.7) is achievable.

**Conclusion:**

Key modifiable risk factors contribute significantly to non-EBF in Nigerian women. Community-based initiatives and appropriate socio-economic government policies that specifically consider those modifiable risk factors could substantially reduce non-EBF practice in Nigeria.

## Background

Non-exclusive breastfeeding (non-EBF) is a risk factor for a number of diseases, including infant mortality from diarrhoea, upper respiratory infections and other common infectious diseases [1–3]. Nigeria – a developing country with 40 million children and the leading beneficiary of developmental assistance for health in Africa [4] – has one of the highest rates of non-EBF among infants aged 0–5 months (84% on average between 1999 and 2013) [5, 6]. This is despite the introduction and implementation of various national and subnational initiatives to reduce non-EBF practice [5, 7]. The Global Burden of Diseases, Injuries, and Risk Factors study 2016 (GBD 2016) ranked non-EBF among the top ten risk factors for under-five deaths and disability-adjusted life years (DALYs) in Nigeria, accounting for 25,300 under-five deaths and 2.1 million DALYs lost in 2016, respectively [8, 9]. This study also found that a large proportion of childhood wasting and malnutrition can be attributed to diarrhoea and other common infectious diseases, which also reflects the role of non-EBF in childhood morbidity.

In Nigeria, various facility- and population-based studies have identified key modifiable factors associated with non-EBF, including socio-economic factors (i.e., low maternal education and poor household wealth) and health service factors (i.e., fewer antenatal care visits, home delivery and delivery assisted by non-health professionals) [5, 10–15]. Additional factors identified in Nigeria include a lack of family support, mother’s employment, and myths and belief systems held for breastfeeding [16–18]. At the population level, the effect of a particular risk factor is dependent on the strength of association of the risk factor with a given outcome, and the prevalence of the risk factor in the population of interest [19, 20]. Changes in the prevalence of non-EBF in Nigeria may be affected by a range of strategic initiatives, including policy development (such as socio-economic reforms to reduce poverty and improve female education); facility-based interventions (such as strengthening the baby friendly hospital initiative) and community-based initiatives (such as the baby friendly community initiative and involvement of family members in infant feeding counselling) [5, 10].

The population attributable fraction (PAF) is the proportional reduction in the incidence of a disease or outcome over a specified time interval that would be achieved by eliminating the exposure(s) of interest from the population under an alternative or counterfactual, more favourable distribution of risk factors [21]. Thus, the PAF measures the proportion of a given health outcome attributable to an exposure in the study population and the hypothetical reduction if the exposure prevalence could be reduced to zero. The PAF also assumes, that the exposure is related to the outcome; measurement of the risk prevalence and association is unbiased and the reduction of the exposure will have no effect on the distribution of other risk factors [21, 22]. The PAF of risk factors for a range of diseases and/or outcomes (including breast cancer, diabetes and child mortality) has been measured globally [23, 24] and for many countries, including Australia [19], United States of America [25, 26], and Nigeria [27].

From a public health viewpoint, estimation of the PAF is of most relevance when the exposure is causally related to the outcome and the factor is amenable to strategic initiatives [21]. Based on nationally representative population-based data, the strongest modifiable risk factors (in terms of the magnitude of effect size) associated with non-EBF in Nigeria include: (i) low or no maternal education, (ii) middle or poor household wealth, (iii) a lack of antenatal care visits, (iv) delivery at home, and (v) delivery assistance from a non-health professional [5, 10]. In Nigeria, no published studies have assessed the attributable risk of important modifiable risk factors associated with non-EBF, nor has there been an investigation of scenarios based on feasible impact of strategic community-based initiatives in reducing exposure prevalence. Thus, this study aims to quantify and compare the burden of non-EBF attributable to key modifiable risk factors in Nigeria to inform strategic policy responses and initiatives.

## Methods

### Data sources

Relative risks and prevalence data used to calculate the PAF for selected modifiable risk factors were based on an analysis of nationally representative data, the Nigeria Demographic and Health Survey (NDHS) for the years 1999 (*N* = 8199), 2003 (*N* = 7620), 2008 (*N* = 33,385) and 2013 (*N* = 38,948). The surveys were conducted by the National Population Commission (NPC) and Inner City Fund (ICF) International using a multi-stage stratified sampling technique. Sample sizes were selected from the 1991 (1999 and 2003 NDHS) and 2006 (2008 and 2013 NDHS) census frames. The increase in sample size in 2008 and 2013 reflects growth in the Nigerian population and an inclusion of additional sets of survey questions. Additional information on the data sources (including data collection techniques) have been described elsewhere [28–30]. We have provided estimates for the period spanning (1999–2013) because (i) the high prevalence of non-EBF has remained unchanged between 1999 and 2013, despite the introduction and implementation of infant and young child feeding policies [6]; (ii) the period captures a time of stable political environment in Nigeria, after more than a decade of authoritarian regimes; and (iii) this study provides additional evidence on strategic initiatives to reduce rate of non-EBF in Nigeria, consistent with previous reports [31, 32]. The stable political system in Nigeria was associated with an increase in health care financing and heightened maternal and child health programmes [7].

### Outcome variable

Exclusive breastfeeding was defined as infants aged 0–5 months who received breast milk as the only source of nourishment, but allowed oral rehydration solution, drops or syrups of vitamins and medicines [33]. In the analysis, non-exclusive breastfeeding (non-EBF) was the main outcome and was expressed as a dichotomous outcome (that is, respondents who exclusively breastfed were coded as ‘0’ and those who did not were coded as ‘1’).

### Modifiable exposures

The exposures considered in the analysis included a range of socio-economic and health service factors, and the selection of these factors was based on evidence from previously published studies from Nigeria [5, 10].

*Socio-economic risk factors* included the mother’s highest educational level (categorized as no education, primary or secondary and above education) and household wealth index (categorized as poor, middle or rich). The household wealth index was calculated as a measure of household assets such as ownership of transportation devices, ownership of durable goods and household facilities, which was derived from a principal components analysis conducted by the National Population Commission (NPC) and ICF Macro [28, 29] and was used in previous studies [5, 13].

*Health service factors* included the number of antenatal clinic visits (categorized as no antenatal visit, one to three antenatal visit or four and above antenatal visits, reflecting the WHO four-visit ANC model for focused antenatal care) [34] and the place of delivery (categorized as health facility or home). The type of delivery assistance received was assessed, and was categorized as either a health professional, traditional birth attendant or untrained personnel. A traditional birth attendant is usually a woman who assists the mother during childbirth and who initially acquired her skills by delivering babies herself, or by working with other traditional birth attendants [35]. The prevalence estimates of these key modifiable risk factors were used to calculate the PAF.

### Statistical analysis

The total number of non-exclusive breastfeeding cases were examined over the study period (1999–2013), stratified by socio-economic and health service variables to determine the absolute number of cases of non-EBF within the study population. Prevalence estimates, and calculation of standard errors (for calculation of 95% confidence intervals) were adjusted using sampling weights to account for the cluster sampling design used in the NDHS [28, 29].

Using multi-level regression models to estimate relative risk, relative differences between study factors were investigated over the study period (1999–2013) [Table 1]. Study variables included socioeconomic factors (maternal education and household wealth index) and health service factors (place of delivery, frequency of antenatal visits and delivery assistance). Multi-level regression models adjusted for the potential confounding factors of geopolitical region, maternal age, birth interval and sex of the baby as employed in previous studies [5, 10]. Period was specified in the analyses as a categorical variable, and to reduce recall bias, the analyses were restricted to the youngest living children aged less than 24 months, living with mothers (aged 15–49 years). Multi-level adjustments to estimate the relative risk were conducted using generalized linear latent and mixed models (gllamm) method to account for clustering of individuals within geographic areas [36]. All analyses were carried out in Stata version 13.0 (Stata Inc., College Station, TX, USA) with weighted prevalence estimates calculated using the ‘svy’ function to allow for cluster sampling.

**Table 1:** Relative risk, exposure prevalence for PAF, PIF and estimated cases of non-EBF due to the exposures in Nigeria, 1999–2013

Exposure		Cases /Total cases	Non-cases (controls)/Total controls	Relative Risk (95% CI)	Exposure Prevalence (a) (95% CI)	Estimated prevalence following intervention (a) (95% CI)
Maternal education	Secondary and above education	1669/5891	597/1087	1.00	54.9 (51.9–57.9)	54.9 (51.9–57.9)
	Primary education	1192/5891	237/1087	1.32 (1.08–1.61)	21.8 (19.4–24.4)	22.9 (20.4–25.5)
	No education	3030/5891	253/1087	1.96 (1.56–2.47)	23.3 (20.8–25.9)	22.2 (19.7–24.8)
Household wealth	Rich	980/5891	336/1087	1.00	32.2 (29.4–35.1)	32.2 (29.4–35.1)
	Middle	2364/5891	462/1087	1.30 (1.07–1.58)	44.2 (41.2–47.3)	44.2 (41.2–47.3)
	Poor	2366/5891	246/1087	1.79 (1.42–2.27)	23.6 (21.0–26.3)	23.6 (21.0–26.3)
Antenatal care visits	4+	2515/5891	711/1087	1.00	65.4 (62.6–68.3)	73.9 (71.2–76.5)
	1–3	846/5891	133/1087	1.11 (0.89–1.39)	12.2 (10.4–14.3)	20.7 (18.3–23.3)
	None	2522/5891	243/1087	1.42 (1.16–1.73)	22.4 (19.9–25.0)	5.3 (4.1–6.8)
Place of delivery	Health facility	1902/5891	649/1087	1.00	59.7 (56.7–62.6)	65.8 (62.9–68.6)
	Home	3987/5891	438/1087	1.59 (1.33–1.90)	40.3 (37.4–43.3)	34.2 (31.4–37.1)
Delivery assistance	Health professionals	1876/5891	630/1087	1.00	58.0 (55.0–60.9)	64.2 (61.3–67.1)
	Traditional birth attendants	1325/5891	114/1087	1.95 (1.42–2.67)	10.4 (8.7–12.5)	8.9 (7.3–10.8)
	Untrained personnel	2687/5891	344/1087	1.32 (1.03–1.69)	31.6 (28.9–34.5)	27.0 (24.3–29.7)

Cases: cases of non-EBF; non-cases (control): cases of EBF

(a) Proportion is percent of exposed non-cases, an estimate of the exposure in the population. That is, non-cases as a proportion of total non-cases

*PAF*: Population attributable fraction; *PIF*: Potential impact fraction

For the calculation of the PIF: assumption of continued improvements in high-school completion rates in women [39] was made; impact fractions for maternal education were estimated assuming a 5% relative decrease in the proportion of women not completing high school from 23% to 22%. For antenatal visits, impact fractions were estimated assuming a reduction of 17% in women having no antenatal care (from 22% to 5%) [31, 32]. For delivery assistance and place of delivery, impact fractions were estimated assuming a relative reduction of 15% based on community-based interventions to improve exclusive breastfeeding practice [31]. No alternative scenario was defined for household wealth because of a lack of data relating to interventions resulting in income re-distribution

In this study, the population attributable fraction (PAF) describes the proportion of non-EBF in Nigeria that could hypothetically be prevented if the modifiable risk factors were reduced in the population. Information on risk factor prevalence and relative risk (RR) associated with non-EBF was used to calculate the PAF using the formula [19]:

$$ \mathrm{PAF}=\frac{\sum P\left(\mathrm{RR}-1\right)}{\left(1+\sum P\left(\mathrm{RR}-1\right)\right)} $$

Where *P* is the prevalence of the exposure in the population for a given exposure category, and RR the corresponding relative risk for the exposure category, calculated from the NDHS datasets (1999–2013). Risk factors used in this analysis were low maternal education, poor household wealth, no antenatal clinic visits, home delivery and delivery assistance from untrained personnel. A joint PAF across all risk factors was also calculated using the formula [37]:

$$ \mathrm{PAF}\ \left(\mathrm{combined}\right)=1-\prod \limits_{r=1}^R1-\mathrm{PAF}r $$

Where *r* represents each exposure variable. The assumption that exposures are independent and uncorrelated has been diminished with the use of relative risks that have been adjusted for potential confounders.

Avoidable burden refers to the potential reduction in future burden of disease or health outcome that could be attained by changing the current distribution of risk factors to an alternative distribution of risk factors. Consistent with the classification of the counterfactual distribution of exposure suggested by Murray and Lopez [38], a series of scenarios based on evidence from community interventions to improve exclusive breastfeeding [31, 32] and trends in maternal educational achievement [39] were also used to estimate a feasible minimum risk for each exposure to calculate potential impact fractions [40, 41].

Murray and Lopez [38] classified counterfactual exposure distribution into: (i) *theoretical* minimum risk; (ii) *plausible* minimum risk; (iii) *feasible* minimum risk; and (iv) *cost-effective* minimum risk. Theoretical minimum risk refers to the exposure distribution that would result in the lowest population-level risk, regardless of whether currently achievable. Plausible minimum risk is the imaginable distribution of risks that would reduce the risk in the population of interest, if attained. Feasible minimum risk refers to a distribution of risk that has been achieved in some population, while cost-effective minimum risk reflects the exposure reduction using a range of cost-effective strategic interventions [38, 42]. As noted above, the PAF provides estimates based on the unrealistic counterfactual scenario of the elimination of the exposure from a given population. In this study, potential impact fractions employed estimation of the avoidable burden of non-EBF using a feasible minimum risk distribution, that is, a scenario that could be achieved in a Nigeria based on previous evidence. Accordingly, the present study also estimates potential impact fractions for comparisons with the PAF estimates and to provide evidence on the potential impact of different interventions for policy makers in Nigeria using the formula [43]:

$$ \mathrm{Potential}\ \mathrm{Impact}\ \mathrm{Fraction}\ \left(\mathrm{PIF}\right)=\frac{\sum_c^n=1\ {P}_c{RR}_c-{\sum}_c^n=1\ {P}_c^{\ast }{RR}_c}{\sum_c^n=1\ {P}_c{RR}_c} $$

Where *P*_*c*_ is the proportion of the population in a given exposure category *c*, *RR*_*c*_ is the relative risk for that exposure category calculated from the NDHS dataset relative to the unexposed category, and *P*_c_^*^ is the estimated proportion of the population in a given exposure *c* after the intervention. In this analysis, the estimated effect of interventions was based on previously published studies.

For antenatal visits, impact fractions were estimated assuming a reduction of 17% in women having no antenatal care (from 22% to 5%) [31, 32]. For delivery assistance and place of delivery, impact fractions were estimated assuming a relative reduction of 15% based on community-based interventions to improve exclusive breastfeeding practice [31]. Finally, assuming continued improvements in high-school completion rates in women [39], impact fractions for maternal education were estimated assuming a 5% relative decrease in the proportion of women not completing high school from 23% to 22%. No alternative scenario was defined for household wealth because of a lack of data relating to interventions resulting in income re-distribution.

Monte-Carlo simulation models using Ersatz Software 1.31 [44] were used to estimate the 95% confidence intervals for population attributable fraction and potential impact fraction estimates, to account for the uncertainty around the exposure prevalence and relative risk estimates. A beta probability distribution was used for exposure prevalence estimates (based on cases and non-cases) using the ErBeta function, and a normal distribution was used for relative risk estimates (based on a normal distribution for the natural logarithm of the RR) using the ErRelative Risk function, to estimate 95% confidence intervals for estimates after 10,000 iterations to ensure model convergence. To obtain the number of non-exclusive breastfeeding cases attributable to each exposure, attributable fractions were multiplied by the estimated total number of non-EBF cases.

### Ethics

The DHS project obtained the required ethical approvals from the National Health Research Ethic Committee (NHREC) in Nigeria before the surveys were conducted (Assigned Number NHREC/01/01/2007). Participants were informed of the rationale for the surveys, confidentiality of their responses, and that respondents did not need to answer the questions if they do not feel comfortable doing so. Participants provided written informed consent before they participated in the surveys. The data used in this study were anonymous and publicly available to apply for online. Approval was sought from MEASURE DHS/ICF International and permission was granted for this use.

## Results

A total sample (*N* = 34,653) of maternal responses of infants aged 0–5 months in relation to EBF were examined in Nigeria for the period (1999–2013). More than three quarter (84.4%) of infants 0–5 months of age were not exclusively breastfed. That is, infants who received other water-based liquids in addition to breast milk [Table 1]. The analysis showed that 22.8% (95% CI: 9.2–37.0%) of all estimated cases non-EBF in Nigeria could be attributed to maternal primary level of education and no maternal education [Table 2]. Approximately 25.0% (95% CI: 9.5–39.5%) of all estimated cases of non-EBF in Nigeria for the years 1999–2013 was attributable to lower household wealth. For antenatal care visits, 9.7% (95% CI: 1.7–18.1%) of all cases of non-EBF was attributable to fewer numbers (1–3) of antenatal care visits and no antenatal care visits of Nigerian mothers. Of the estimated cases of non-EBF, 18.8% (95% CI: 6.9–30.8%) was attributable to home birthing. Similarly, 16.6% (95% CI: 3.0–31.3%) of non-EBF in Nigeria was attributable to delivery assistance from non-health professionals (traditional birth attendants and untrained health personnel).

**Table 2:** PAF and PIF for selected modifiable exposures associated with non-EBF in Nigeria (1999–2013)

Risk factors	Cases of non-EBF (a)	PAF% (95% CI)	PIF% (95% CI)
Low and no maternal education	4222	22.8 (9.2–37.0)	0.5 (−7.0; 7.7)
Middle and poor household wealth	4730	24.7 (9.5–39.5)	–
Lower number (1–3) and no antenatal care visits	3368	9.7 (1.7–18.1)	5.4 (−3.0; 13.9)
Home delivery	3987	18.8 (6.9–30.8)	2.8 (−3.6; 8.9)
Delivery assistance from non-health professional (traditional birth attendants and untrained personnel)	4012	16.6 (3.0–31.3)	2.3 (−4.6; 9.2)
Joint PAF and PIF combinations (in descending order)	Cases of non-EBF (a)	Joint PAF% (95%CI)	Joint PIF% (95%CI)
Mat. Edu + H. wealth + ANC + Pl. delivery + Del. assistance	20,319	64.5 (50.5–76.2)	–
Mat. Edu + H. wealth + ANC + Pl. delivery	16,307	57.4 (42.4–70.2)	–
Mat. Edu + ANC + Pl. delivery + Del. assistance	15,589	52.8 (37.4–66.4)	10.5 (−5.4; 24.7)
Mat. Edu + H. wealth + ANC	12,320	47.8 (31.2–61.7)	–
Mat. Edu + H. wealth	8952	41.9 (25.3–56.9)	–
ANC + Pl. delivery + Del. assistance	11,367	38.9 (23.5–53.2)	8.0 (−2.2;17.9)
Maternal education	4222	22.8 (9.1–37.4)	–

(a) Cases of non-EBF estimated from the total sample

*PAF*: Population attributable fraction; *PIF*: Potential impact fraction; Potential impact fractions calculated only for exposures where published intervention estimates were available. Mat. Edu: low and no maternal education; H. wealth: middle and poor household wealth; ANC: lower number (1–3) and no antenatal care visits; Pl delivery: home delivery; Del. assistance: delivery assistance from a non-health professional (traditional birth attendants and untrained personnel)

In combination, the joint PAF showed that 64.5% (95% CI: 50.5–76.2%) of all cases of non-exclusive breastfeeding in Nigeria for the years 1999–2013 could be attributed to the modifiable risk factors of low or no maternal education, poor or medium household wealth, lower frequency of antenatal care visits, home delivery and delivery assistance from non-health professionals (Table 2). Key socioeconomic indicators (i.e., low or no maternal education and poor or middle household wealth) accounted for the largest PAF of non-EBF in Nigeria, followed by the health service factors (home delivery and fewer/no antenatal care visits).

Potential impact fractions assuming improvements in maternal education [39], the number of antenatal visits, and a higher proportion of deliveries in a health facility with increased training of health professionals [31, 32] suggested that 10.5% (95% CI: -5.4; 24.7) of cases of non-EBF could be avoided in Nigeria (Table 2). This included 0.5% (95% CI: -7.0; 7.7) associated with a 5% relative increase in the number of women finishing secondary school; 5.4% (95% CI: -3.0; 13.9) associated with a 17% reduction in the proportion of women who had less than four antenatal care; and 2.8% (95% CI: -3.6; 8.9) and 2.3% (95% CI: -4.6; 9.2) associated with a 15% decrease in the proportion of deliveries occurring outside a health facility, with untrained personnel and traditional birth attendants, respectively.

## Discussion

This study estimated the number of cases of non-EBF in Nigerian mothers attributable to the key modifiable risk factors of maternal education, household wealth, antenatal visits, home delivery, and delivery assistance from a non-health professional. The largest population attributable fraction was associated with no maternal education, followed by poor household wealth, home delivery and delivery assistance from non-health professionals. Assuming similar impacts of published community-based interventions in improving health service contacts of Nigerian women [31, 32] and continued improvements in maternal education [39]; this study also suggests that approximately 11% of cases of non-EBF could potentially be avoided in Nigeria.

This study has a number of limitations. First, non-EBF practice was based on self-report and this could result in measurement bias as mothers may incorrectly recall how the baby was fed in the period referred to in the surveys. Nonetheless, attempts were made to reduce recall bias in the survey through the use of standardised questionnaires, and in the analyses by restricting analyses to the youngest living child aged < 24 months living with the mother, consistent with previous studies [45, 46]. Second, misclassification bias in the exposure variables may also have occurred – for example, underestimation or overestimation of the number antenatal care visit – which may result in overestimation or underestimation of the association between antenatal care visits and non-EBF.

Third, the study was based on cross-sectional data, and the establishment of a clear causal relationship between exposure variables and non-EBF practice is difficult. However, the associations reported in this study were consistent with previously published studies from other developing countries which used nationally representative prevalence data (Demographic and Health Survey data) [47, 48]. Additionally, it is possible that the data reflected in this analysis may not represent the most current situation in Nigeria given changes in the socio-demographic and economic status [49].

Fourth, the burden of non-EBF attributable to key modifiable risk factors (examined in this study) was based on nationally representative data; however, this does not take into account other modifiable factors associated with non-EBF (for example, mothers employment status, a lack of family support and socio-cultural belief systems held for breastfeeding) as reported in published studies from regional areas of Nigeria [16, 17, 50].

Fifth, unmeasured confounding factors (such as maternal health problems in the early postnatal period or twin birth) are also likely to affect the study findings. The wealth index used in the NDHS is an indirect measure of household wealth, since it is challenging to obtain reliable income and expenditure data in Nigeria.

Sixth, there are no adequate annual data on non-EBF in Nigeria, nor is there contemporaneous data on relevant breastfeeding policy implementation and evaluation in order to estimate the PIF with the prevalence of non-EBF. Information on annual non-EBF practice and long-term breastfeeding policy implementation and evaluation would have provided a specific representation on the avoidable of cases of non-EBF in the present Nigeria.

Seventh, the applicability of the potential impacts of community-based interventions in the Nigerian context may be challenging given the influence of geopolitical differences on infant and young child feeding practices in Nigeria [10]. However, both population attributable fractions and potential impact fractions were presented in this study to provide an indication of the range of feasible impacts of key community- and facility-based interventions to reduce non-exclusive breastfeeding practice in Nigeria.

Despite these limitations, the study has a number of strengths. Firstly, the prevalence data used (NDHS, 1999–2013) are nationally representative and provide important information on infant feeding practices (including risk factors categories) that could be used to estimate relative risks, as previously reported [19, 51, 52]. Secondly, selection bias is unlikely in the data as samples were drawn from the 1991 and 2006 national census frames, with response rates in the surveys ranging from 92 to 98%. Additionally, the risk factors assessed in this study are amenable to strategic policy responses and initiatives as suggested by Rockhill et al. [21], and this study provides context-specific evidence on interventions that can potentially reduce non-EBF practice in Nigeria using summary population health metrics that have been used in a number of previously published studies [19, 24, 51, 53, 54]. For example, evidence from some of these studies, including the Global Burden of Diseases, Injuries, and Risk Factors Study, have been used to describe and prioritise key health initiatives and policies in many countries, including the United Kingdom, Australia, Rwanda, South Africa, United States of America and Columbia [55].

Evidence from the current study showed that more than half (65%) of all cases of non-EBF in Nigeria are attributable to a range of modifiable risk factors amenable to public health initiatives and socio-economic reforms – areas where Nigeria receives substantial support from the international community in terms of funding [4]. For example, in 2015, the World Bank approved USD500, 000 million for Nigeria to provide assistance for the “saving one million lives” project – an initiative to improve key areas of maternal and child health, including nutrition [56]. A similar large-scale public health intervention conducted in Bolivia, Ghana and Madagascar (funded by an international donor) resulted in a significant increase in not only exclusive breastfeeding, but also improvements in early initiation of breastfeeding [31]. As the Nigerian government and donor agencies work toward the achievement of this “saving one million lives” project, the Global Nutrition Targets [57] and the Sustainable Development Goals (SDGs) in Nigeria; considerable improvements in child nutrition and health can be made if specific and measureable objectives are set towards reducing these observed modifiable risk factors in the Nigerian context to reduce non-EBF.

Additional modifiable risk factors associated with non-exclusive breastfeeding in Nigeria not examined in this study (because of a lack of data) include: poor family support, pressure on the mother to resume work, and myths and belief systems held for breastfeeding; for example, the infant is perceived to still be hungry after breastfeeding [16, 17] or the baby requires additional water to quench thirst [58]. Although these risk factors were not assessed, large scale community- and facility-level initiatives that specifically target the modifiable risk factors examined in this study can also reduce the unassessed risk factors associated with non-EBF in Nigeria as reported in previous studies [31, 32]. Another important factor that may be problematic to measure is political context, with studies suggesting that a high-level of political will and commitment plays a major role in reducing non-EBF in other context [32]. Given the current health initiatives in Nigeria and increasing international funding for maternal and child health initiatives [4], sustained political resolve at all levels is also needed to achieve substantial reduction in non-EBF in Nigeria.

Geopolitical region, culture and religion play major roles in policy formulations in Nigeria – a country that is largely divided into Muslim-north and Christian-south [59, 60]. Accordingly, strategic interventions and policies responses that are designed to reduce non-exclusive breastfeeding practice in Nigeria should be context-specific, and must consider the impacts of geopolitical differences, culture and religion on optimal infant and young child feeding practices in Nigeria. A detailed description of locally-relevant interventions and policies to improve EBF in Nigeria has been reported elsewhere [6]. These include stronger political will and funding, strengthening community and facility-based participation, and refining standalone/integrated infant and young child policy implementations.

## Conclusion

This study focussed on key modifiable risk factors that have previously been associated with non-EBF in Nigeria. The estimated attributable burden of each of the modifiable risk factors was substantial. For the combined PAF, more than half of all estimated cases of non-EBF among infants aged 0–5 months in Nigeria can be attributed to the four risk factors examined. Substantial reduction in the prevalence of non-EBF in Nigeria is achievable if initiatives particularly target low socio-economic mothers, and those who have few contacts with health services.
